# Imaging Surveillance Adherence After Endovascular Abdominal Aortic Aneurysm Repair at VA Hospitals

**DOI:** 10.1001/jamanetworkopen.2025.6852

**Published:** 2025-04-24

**Authors:** Laura E. Newton, Aravind Ponukumati, Gabrielle Zwain, Caroline Korves, Jialin Mao, Kayla Moore, Shipra Arya, Olamide Alabi, Salvatore Scali, Erin Greenleaf, David Stone, Emily Spangler, Philip Goodney

**Affiliations:** 1 Department of Surgical Services, Veterans Affairs Medical Center, White River Junction, Vermont; 2 Department of General Surgery, Dartmouth Health, Lebanon, New Hampshire; 3 Department of Vascular Surgery, Dartmouth Health, Lebanon, New Hampshire; 4 Clinical Epidemiology Program, Veterans Affairs Medical Center, White River Junction, Vermont; 5 Department of Population Health Sciences, Weill Cornell Medicine, New York, New York; 6 Division of Vascular Surgery, Stanford University School of Medicine, Palo Alto, California; 7 Section of Vascular Surgery, Veterans Affairs Palo Alto Healthcare System, Palo Alto, California; 8 Department of Vascular Surgery and Endovascular Therapy, Emory University Hospital, Atlanta, Georgia; 9 Surgical and Perioperative Services, Atlanta Veterans Affairs Healthcare System, Atlanta, Georgia; 10 Division of Vascular Surgery and Endovascular Therapy, University of Florida, Gainesville; 11 Division of Vascular Surgery, Malcom Randall Veterans Affairs Medical Center, Gainesville, Florida; 12 Division of Vascular Surgery and Endovascular Therapy, Department of Surgery, Baylor College of Medicine, Houston, Texas; 13 Department of Surgery, Michael E. DeBakey Veterans Affairs Hospital, Houston, Texas; 14 Department of Surgery, Division of Vascular Surgery and Endovascular Therapy, University of Alabama at Birmingham; 15 Department of Surgery, Birmingham Veterans Affairs Medical Center, Birmingham, Alabama

## Abstract

**Importance:**

Guidelines recommend annual imaging surveillance after endovascular abdominal aortic aneurysm repair (EVAR). How these guidelines translate into practice among veterans remains poorly described.

**Objective:**

To characterize post-EVAR surveillance among veterans.

**Design, Setting, and Participants:**

This retrospective cohort study evaluated veterans who underwent EVAR between January 1, 2000, and December 31, 2023, at US Department of Veterans Affairs (VA) hospitals and received follow-up care at VA and non-VA hospitals and imaging centers with payment via Medicare or the VA. Veterans treated with EVAR in VA hospitals during the study period were included.

**Exposure:**

Years after EVAR.

**Main Outcomes and Measures:**

The primary outcome was annual surveillance adherence, measured as 1 or more imaging studies in the abdomen or pelvis each year after EVAR. Stepwise logistic regression modeling was used to determine factors associated with poor adherence. Secondary outcomes were imaging type (cross-sectional, ultrasonography, or ultrasonography followed by cross-sectional imaging) and factors associated with lower adherence.

**Results:**

The cohort included 27 792 veterans (27 624 male [99.4%]; 22 521 aged ≥65 years [81.0%]). Mean (SD) follow-up was 6.0 (4.0) years. The mean (SD) proportion of time that veterans were surveillance adherent was 71.1% (28.5%). Surveillance was initially high, with 25 026 of 27 792 veterans (90.0%) undergoing surveillance imaging in year 1 after EVAR. However, this proportion decreased further out from EVAR, with 12 401 of 21 384 veterans (58.0%) undergoing surveillance imaging by year 4 after EVAR. Veterans were most likely to undergo imaging with computed tomography scans (21 911 veterans [78.8%]). However, the proportion with surveillance via ultrasonography alone increased from 823 of 25 026 veterans (3.3%) in year 1 after EVAR to 2567 of 12 401 veterans (20.7%) in year 4 after EVAR. White race (odds ratio [OR] vs all other racial groups, 0.84; 95% CI, 0.72-0.98), married status (OR vs all other social status categories, 0.80; 95% CI, 0.71-0.89), having a service-connected disability (OR, 0.69; 95% CI, 0.62-0.77), and a higher Charlson Comorbidity Index score (OR per 1-unit increase, 0.93; 95% CI, 0.91-0.95) were associated with lower odds of poor surveillance adherence.

**Conclusions and Relevance:**

In this study, post-EVAR imaging surveillance was high, although surveillance lapses were more likely further out from EVAR and for patients with certain characteristics. This information may inform future patient-centered efforts to improve post-EVAR imaging adherence.

## Key Points

**Question:**

Do veterans treated with endovascular abdominal aortic aneurysm repair (EVAR) at VA hospitals receive guideline-adherent annual postoperative surveillance imaging?

**Findings:**

In this cohort study of 27 792 veterans, the mean proportion of time veterans were adherent with surveillance recommendations over their postoperative lifetime was 71.1%. This was time dependent, with 90.0% and 58.0% of veterans undergoing surveillance in year 1 and year 4, respectively.

**Meaning:**

This study found that post-EVAR imaging surveillance among veterans was guideline adherent for more than 70% of veterans’ postoperative lifetime.

## Teaser

This cohort study investigates rates of guideline-adherent annual imaging surveillance after endovascular abdominal aortic aneurysm repair (EVAR) among patients at Veterans Affairs hospitals.

## Introduction

Elective repair of abdominal aortic aneurysms (AAAs) effectively mitigates their risk of rupture.^1,2^ When AAA anatomy can accommodate an aortic endograft, endovascular aneurysm repair (EVAR) is often favored given low rates of early postoperative complications, lower in-hospital mortality, and shorter recovery time compared with open surgical repair.^2,3,4,5,6^ However, patients and surgeons must weigh these early benefits against increased aortic reinterventions after the initial repair and the need for lifelong annual surveillance.^7,8^ Guidelines and recommendation from the Society for Vascular Surgery (SVS), American Heart Association, and Food and Drug Administration (FDA) all recommend annual follow-up imaging after EVAR.^2,9,10^ Current SVS guidelines recommend a baseline contrast-enhanced computed tomography (CT) scan and color duplex ultrasonography (US) within 30 days after EVAR. If no endoleak or sac enlargement is detected, repeat imaging should occur at 1 year after EVAR and annually thereafter using CT or US imaging.^2^

Despite these recommendations, prior studies have demonstrated that as many as 60% of patients treated with EVAR experience gaps in surveillance, particularly farther out from their procedure.^11,12,13^ Additionally, the imaging modality used for surveillance can vary relative to physician preferences and patient comorbidities. While the most recent guidelines suggest that color duplex US be used more frequently in the absence of endoleak or aneurysm expansion, the extent to which US has replaced CT in routine practice is unknown. Many gaps in surveillance have been shown to depend on insurance access, variation in resources available, and how care is provided at individual centers.^14^ Overall, the translation of imaging surveillance guidelines into clinical practice and how patient factors may be associated with this process remain incompletely characterized.

Veterans receiving vascular care in US Department of Veterans Affairs (VA) hospitals have insurance for longitudinal surveillance studies and a robust quality assessment system to monitor postoperative surgical care and surveillance. We hypothesized that veterans treated with EVAR offer a robust opportunity for studying long-term process and outcome measures, such as follow-up after surgical procedures. This study aims to characterize longitudinal surveillance imaging after EVAR, describe the types of imaging used over time, and determine factors associated with lapses in surveillance.

## Methods

This cohort study was approved by the VA Central Institutional Review Board, which provided a waiver of informed consent because of the minimal risk posed to participants and because the retrospective nature of the study made it impractical to conduct without the waiver. The Strengthening the Reporting of Observational Studies in Epidemiology (STROBE) reporting guideline was used in the conducting and reporting of this study.

### Data Sources

This retrospective cohort study evaluated veterans who underwent EVAR between January 1, 2000, and December 31, 2023, in VA hospitals. Electronic health records were used from the Veterans Health Administration (VHA) Corporate Data Warehouse (CDW), Centers for Medicare & Medicaid Services (CMS) through the VA Information Resource Center, and Community Care data, including Integrated Veteran Care (IVC) Consolidated Data Sets (CDS), Purchased Care (Fee), and the Archived Program Integrity Tool (PIT).

### Identification of Cohort of Veterans Treated With EVAR

The cohort included veterans aged 19 years or older with AAAs identified by relevant *International Classification of Diseases, Ninth Revision *(*ICD-9*) and *International Statistical Classification of Diseases and Related Health Problems, Tenth Revision *(*ICD-10*) codes, *ICD-9* and *ICD-10* procedure codes, and *Current Procedural Terminology* (*CPT*) codes from January 1, 2000, to December 31, 2023. We used the VA Phenomics Library–Centralized Interactive Phenomics Resource to find best practices and standard operating procedures in identifying appropriate *CPT* and *ICD-9* and *ICD-10* codes (eTable 1 in Supplement 1).^15^ Veteran demographics and each veteran’s first EVAR were identified using *ICD-9* and *ICD-10* procedure codes and *CPT* codes in CDW data only. Race and ethnicity were also identified from VA electronic health records databases in CDW. Race and ethnicity categories in CDW included American Indian or Alaska Native, Asian, Black, Native Hawaiian or Pacific Islander, and unknown (declined to state or unknown). Hispanic ethnicity categories included Hispanic or Latino, not Hispanic or Latino, declined to answer, and unknown by patient. Race and ethnicity were included in this assessment to understand the demographics of the cohort and how patient factors may be associated with surveillance patterns after EVAR. Veterans were included whether the EVAR was elective, urgent, or emergent. Nonveterans, individuals who underwent open AAA repair before their first identified EVAR, and those with missing or unreliable demographic data (such as a missing date of birth or a date of death preceding EVAR) were excluded. Index EVAR procedures were limited to those that occurred at the VA, but exclusionary events (ie, open AAA repair and other EVAR events outside of the VA) included data from CMS and Community Care.

Regarding geographic variables, the Planning Systems Support Group (PSSG) was used to determine each veteran’s drive time between the home address and the nearest tertiary care VA facility.^16^ This file has data from 2009 to 2022 and includes drive distance and times from the patient’s home address as enrolled to the nearest primary care, secondary care, and tertiary care facilities. Patient addresses are updated quarterly using the VHA Assistant Deputy Under Secretary for Health Enrollment File and the US Postal Service National Change of Address database. The estimated driving time and distance is based on an enrollee’s geocoded residential location to the nearest VA sites. Drive time data that were temporally closest to each veteran’s EVAR procedure date were used. For example, for veterans with an EVAR procedure date before 2009, we used 2009 PSSG drive time data; for veterans with an EVAR procedure after 2009, we used PSSG drive time data for that EVAR year or for 2022.

### Identification of Surveillance Imaging Studies

We identified surveillance imaging studies using *CPT* codes in the following sources: CDW in the form of outpatient procedures, inpatient *CPT* procedure codes, radiology exams, and consults^15,17^; CMS in the form of outpatient revenue, inpatient revenue, and carrier files; and Community Care in the form of fee service provided, fee initial treatment, IVC CDS claim line, IVC CDS claim line procedure, PIT claim, and PIT claim details^15^ (eTables 2-3 in Supplement 1). Surveillance imaging studies were included if they occurred on or after the first identified EVAR until the veteran’s censor date or the end of the study period. Veterans were censored at the first date of enrollment in Medicare Advantage (if occurring after the index EVAR procedure), date of death, or the end of the study period. If a patient was already enrolled in Medicare Advantage at the time of the index EVAR procedure at the VA, the patient was not censored. Only individuals whose index EVAR procedure at the VA preceded their enrollment in Medicare Advantage were censored owing to lack of Medicare Advantage data (2612 individuals). Unique imaging studies were defined as those having a different *CPT* code and date per veteran within the data source.

### Primary Outcome Measure: Surveillance Adherence

The primary outcome was adherence to annual imaging surveillance, defined as having at least 1 imaging study (CT, US, or magnetic resonance imaging [MRI]) per year after EVAR as a minimum threshold of imaging that should occur after EVAR based on SVS guidelines and FDA Instructions for Use documents.^2,10^ A lapse in surveillance adherence was defined as any 12-month period during which a surveillance imaging study was not obtained.

The proportion of time spent in adherence to surveillance was calculated using the number of total 12-month increments after EVAR to the end of follow-up (the first occurrence of the date of death, censoring for Medicare Advantage enrollment, or study period end) and the number of 12-month increments in which an imaging study was obtained (adherent year). For example, this was calculated at the individual patient level using the following formula: No. of 12-month increments with surveillance imaging for the patient/the total No. of 12-month increments from patient EVAR to the end of follow-up. This methodology has been used to calculate adherence in cancer surveillance, vaccine administration, and other longitudinal quality metrics.^18,19,20^ The overall number of imaging studies per veteran per year was also calculated. To limit variation from small sample sizes, we performed a subanalysis including only veterans who had 5 or more years of follow-up data.

### Secondary Outcomes: Types of Surveillance Imaging

The secondary outcome was the proportion of each imaging study type over time. Imaging studies were categorized into 4 types: US alone, US followed by CT within 30 days, CT alone (without evidence of antecedent US), or other, which included MRI.

### Statistical Analysis

Descriptive statistical analyses were performed in SAS Grid Manager Client Utility version 9.46 (SAS Institute). Means with SDs and medians were reported for continuous variables, whereas frequencies and proportions were reported for categorical variables.

We divided veterans into 5 categories of surveillance adherence from lowest to highest (0%-20%, 21%-40%, 41%-60%, 61%-80%, and 81%-100%) and constructed a histogram demonstrating these categories. To determine factors associated with an individual patient’s surveillance adherence, a stepwise logistic regression model was used to identify factors associated with the lowest category of adherence at the patient level. We identified variables for inclusion in the stepwise regression based on differences in baseline characteristics that we observed between groups and factors that we hypothesized would be associated with adherence. These variables were White race, Hispanic ethnicity, married status, age, male sex, rurality,^21^ priority level 1 to 4,^22,23^ service-connected disability,^24^ drive distance to a VA facility (a binary variable based on > or≤50 miles), Quan Charlson Comorbidity Index (CCI) score,^25^ tobacco use, and problem alcohol use. The stepwise model specified an entry *P* value of .25 and an exit *P* value of .10; *P* values were 2-sided.

## Results

### Demographics of the Cohort

Our cohort included 27 792 veterans who underwent EVAR at VA hospitals during the study period (27 624 male [99.4%]; 22 521 aged ≥65 years [81.0%]; 2312 Black [8.3%], 23 003 White [82.8%], and 436 other race [1.6%]; 803 Hispanic [2.9%]) (eFigure 1 in Supplement 1 and Table 1). Additional characteristics of the cohort, including distribution by Veterans Integrated Service Network are given in eTable 4 in Supplement 1. There were 54 veterans who died on their EVAR procedure date and thus were censored at that time.

**Table 1.** Study Population Characteristics

Characteristic	Veterans, No. (%) (N = 27 792)
Age, mean (SD), y	71.7 (7.7)
<65	5271 (19.0)
65-69	7011 (25.2)
70-74	6587 (23.7)
75-80	4726 (17.0)
>80	4197 (15.1)
Sex	
Male	27 624 (99.4)
Female	168 (0.6)
Race	
Black	2312 (8.3)
White	23 003 (82.8)
Other^a^	436 (1.6)
Unknown^b^	2041 (7.3)
Ethnicity	
Hispanic	803 (2.9)
Missing^c^	1572 (5.7)
Rural^d^	11 091 (39.9)
Drive distance to VA facility, mean (SD), miles^e^	84.0 (90.4)
VA priority levels 1-4	8388 (30.2)
Service connected	13 948 (50.2)
Tobacco use^f^	24 803 (89.2)
Problem alcohol use	3627 (13.1)
Married	13 816 (49.7)
Quan CCI score, mean (SD)	3.5 (2.6)
Index EVAR type	
Complex	952 (3.4)
Standard	26 840 (96.6)
No. of years of follow-up, mean (SD)	6.0 (4.0)
Imaging type^g^	
US alone	2216 (8.0)
US followed by CT within 30 d	2458 (8.8)
CT alone	21 911 (78.8)
Other	152 (0.5)
None	1055 (3.8)

Abbreviations: CCI, Charlson Comorbidity Index; CT, computed tomography; EVAR, endovascular abdominal aortic aneurysm repair; US, ultrasonography; VA, Department of Veterans Affairs.

^a^ Other race included American Indian or Alaska Native, Asian, and Native Hawaiian or Pacific Islander.

^b^ Unknown race included declined to state and unknown.

^c^ Missing ethnicity included declined to answer and unknown by patient.

^d^ Defined as rural-urban commuting area codes for highly rural or rural.

^e^ Drive distance from a veteran’s home address to the closest tertiary care VA site; 3853 (13.9%) veterans had missing data for this variable.

^f^ Defined as former or current smoker.

^g^ Imaging type was obtained during each veteran’s first surveillance imaging encounter.

### Imaging Studies Performed Over the Study Period

**Figure 1.**
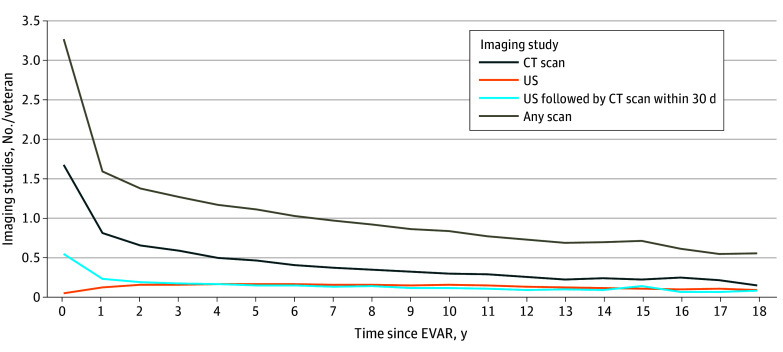
Overall Rate of Imaging Studies The rate of imaging studies is given per veteran per year after endovascular abdominal aortic aneurysm repair (EVAR). CT indicates computed tomography; US, ultrasonography.

We identified a total of 299 390 imaging studies performed on these 27 792 veterans during the study period. Mean (SD) follow-up was 6.0 (4.0) years, ranging from 0 to 24 years. The overall rate of any imaging studies was initially high, at 3.3 studies per veteran during the first 12 months after EVAR (Figure 1). The first time the overall rate decreased to below the recommended annual imaging rate, or 1 scan per veteran per year, was in the eighth year after EVAR. Most images obtained were CT scans (21 911 veterans [78.8%]), with US the next most common imaging type (2216 veterans [8.0%]).

### Primary Outcome: Proportion of Time in Imaging Adherence

**Figure 2.**
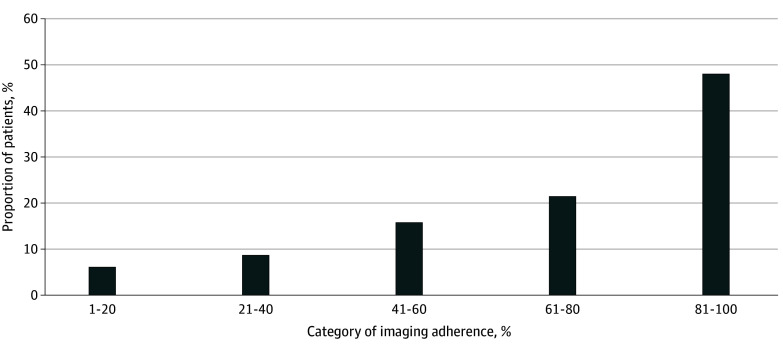
Histogram of Categories of Imaging Surveillance Adherence

The highest category for adherence (80%-100%) included 13 344 veterans (48.0% of the cohort) (Figure 2). The mean (SD) proportion of years a veteran spent in adherence was 71.1% (28.5%) (eTable 5 in Supplement 1). The mean (SD) proportion of years in adherence ranged from 4.6% (6.7%) for individuals in the lowest category of imaging adherence to 95.2% (7.3%) for individuals in the highest category of adherence (eTable 6 in Supplement 1).

Surveillance rates were initially high, with 25 026 of 27 792 veterans (90.0%) undergoing surveillance imaging within their first year after EVAR. However, this decreased to 18 505 of 27 738 veterans (66.7%) in the second year after EVAR and further to 12 401 of 21 384 veterans (58.0%) in the fourth year after EVAR. By year 7 after EVAR, fewer than half of veterans were in adherence (6470 of 13 231 veterans [48.9%]). Among 158 veterans with the longest available follow-up, 45 veterans (28.5%) were adherent in the 20th year after EVAR. Among veterans with a lapse in surveillance, the mean (SD) time to that lapse was 2.9 (2.6) years (eFigure 2 in Supplement 1), and the mean (SD) time until reentry into adherence was 4.1 (2.4) years.

A subanalysis of veterans with 5 or more years of follow-up data demonstrated a mean (SD) proportion of time spent in adherence of 67.6% (25.5%). Among veterans in this subgroup who experienced a lapse in surveillance, the mean (SD) time to that lapse 4.8 (3.0) years.

### Secondary Outcome: Imaging Types Over Time

**Figure 3.**
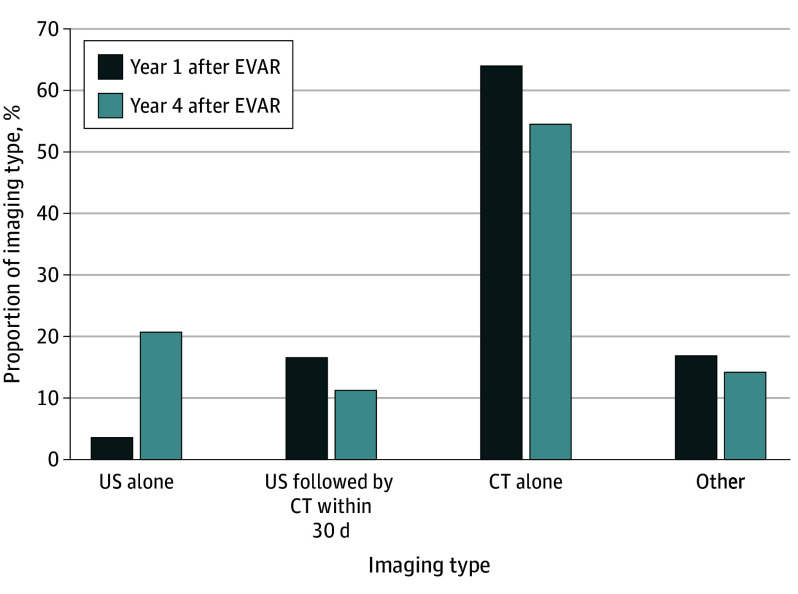
Proportion of Imaging Type by Year Proportions are given by years after endovascular abdominal aortic aneurysm repair (EVAR) and were calculated only among veterans who had surveillance imaging during those years. CT indicates computed tomography; US, ultrasonography.

Veterans followed up with surveillance imaging were more likely to undergo CT scans than any other imaging study (78.8% of veterans). However, with increased time after EVAR, the proportion of veterans undergoing imaging surveillance via CT decreased, from 15 999 of 25 026 veterans (63.9%) in year 1 after EVAR to 6750 of 12 401 veterans (54.4%) in year 4 after EVAR. Conversely, the proportion of veterans undergoing imaging surveillance via US alone increased from 823 of 25 026 veterans (3.3%) in year 1 after EVAR to 2567 of 12 401 veterans (20.7%) in year 4 after EVAR (Figure 3).

### Multivariable Model to Describe Poor Imaging Adherence

Stepwise regression results indicated that White race (OR vs all other racial groups, 0.84; 95% CI, 0.72-0.98), married status (OR vs all other social status categories, 0.80; 95% CI, 0.71-0.89), a service-connected disability (OR, 0.69; 95% CI, 0.62-0.77), and a higher baseline Quan CCI score (OR per 1-unit increase, 0.93; 95% CI 0.91-0.95) were associated with a lower odds of poor adherence to imaging surveillance guidelines (Table 2). A summary of how our study compares to prior work on imaging surveillance after EVAR is found in eTable 6 in Supplement 1.^11,12,13,26,27,28^

**Table 2.** Multivariable Model of Factors Associated With Poor Surveillance Adherence

Factor	OR (95% CI)
White race vs all other racial groups^a^	0.84 (0.72-0.98)
Married vs all other social status categories^b^	0.80 (0.71-0.89)
Service-connected disability vs no service-connected disability	0.69 (0.62-0.77)
Quan CCI score, per 1-unit increase	0.93 (0.91-0.95)

Abbreviations: CCI, Charlson Comorbidity Index; OR, odds ratio.

^a^ Other racial groups included American Indian or Alaska Native, Asian, Black, Native Hawaiian or Pacific Islander, and unknown (declined to state and unknown).

^b^ Other social status categories included single, widow or widower, divorced, never married, unknown, and missing.

## Discussion

In this national cohort study, veterans were adherent to imaging surveillance after EVAR 71.1% of the time. Guideline-adherent surveillance was highest in the first year after EVAR, and rates of adherence decreased in each year thereafter, with the greatest declines in years 2 and 3 after EVAR. Despite recommendations to increase the use of US for annual surveillance after EVAR, more than half of veterans undergoing surveillance still did so via CT.

Imaging surveillance after EVAR has been measured previously in national studies using administrative and registry-based data sources. A study of Medicare beneficiaries^13^ defined incomplete surveillance as intervals of greater than 15 months between consecutive imaging studies and found that 43% of patients had complete surveillance during a median follow-up of 6.1 years. A study of surveillance imaging after EVAR in the Vascular Quality Initiative (VQI)^11^ using Kaplan-Meier survival analysis reported 50% surveillance failure by 4.19 years and 75% failure by 6.35 years. Both studies reported high rates of surveillance imaging in the first year after EVAR, followed by steady decreases in surveillance thereafter, which was similar to the results of this study. However, our analysis examined patterns after a lapse in surveillance, when veterans may or may not have reentered guideline adherence. This allowed for a picture of post-EVAR imaging trends in clinical practice over an individual’s remaining lifetime more than prior studies, which examined the surveillance pattern only up until the first lapse in surveillance. While our study did not directly compare surveillance after EVAR performed in VAs vs those performed for nonveterans in the community, our findings suggest a higher adherence rate among veterans.

In our study, the mean first year for a veteran to exit surveillance adherence was just before 3 years, at year 2.9, which is slightly longer compared with results from prior work in nonveteran populations.^13^ However, a lack of nonveteran data in our analysis prohibits a direct comparison of VA vs non-VA EVAR surveillance. High rates of reintervention with EVAR, up to 5% risk of late rupture, and a reported association between lack of surveillance imaging and increased risk of graft complications prompted the passage of societal and FDA-endorsed guidelines recommending lifelong surveillance after EVAR.^2,8,10,27,29^ However, other study results question whether long-term annual imaging is necessary for all patients and suggest that spacing surveillance intervals up to every 3 years may be safe in certain individuals at low risk.^26,28,30,31^ Furthermore, studies from 2009 to 2021^32,33,34,35^ found that the safety and efficacy of surveillance with US were comparable to those with CT, and several guidelines outside the US, as well as the most recent SVS guidelines, reflect these findings. In this study, more veterans underwent surveillance imaging via US further out from their EVAR, suggesting that more surgeons and patients opt for a less costly and less risky surveillance option as years pass. Additionally, there may be a wider availability of US across geographic areas and care settings. It is unknown whether imaging type is associated with long-term outcomes among veterans, and our future work will assess whether variability in post-EVAR surveillance is associated with survival, reintervention rate, rupture, and contrast-related outcomes, such as acute kidney injury.

### Limitations

Our work has limitations. The available data did not include veterans who underwent EVAR outside the VA. This is important in the age of the Maintaining Internal Systems and Strengthening Integrated Outside Networks (MISSION) Act of 2018, after which there was a reported shift in a portion of procedural care from the VA to the community.^36,37,38^ Additionally, although this analysis included imaging studies performed outside the VA, it is possible that some veterans undergoing surveillance in the community were not captured in our search. Further work is required to characterize trends in imaging surveillance after veterans undergo EVAR in the community. A strength of this study was the more than 20 years of data, with a mean follow-up of 6 years per patient. However, this retrospective observational analysis relies on billing claims to detect clinical events and thus lacks granularity regarding patient-specific considerations as to why surveillance imaging was not obtained, such as limited life expectancy. Furthermore, surveillance imaging studies were defined broadly given that vascular-specific imaging could not be isolated from studies for nonvascular indications. Our geographic data on drive times were limited by reliance on the PSSG, which includes only data from 2009 to 2022. Therefore, for veterans with an EVAR date before 2009 or after 2022, the home address used to calculate drive time may not match where the veteran lived at the time of the EVAR. Additionally, this study is descriptive and characterizes the state of surveillance after EVAR among veterans, but future research is needed to understand the impact of surveillance frequency and modality on outcomes after EVAR.

## Conclusions

This national cohort study of EVAR in VA hospitals over the last 20 years characterizes post-EVAR imaging surveillance rates among veterans. Surveillance rates initially approached guideline adherence, but lapses in adherence remained common, especially among veterans who were not married, were members of racial minority groups, or did not have a service-connected disability. Most veterans underwent cross-sectional imaging studies, although the proportion undergoing US studies increased further out from EVAR. Although we did not conduct a direct comparison, our results suggest a higher rate of post-EVAR surveillance adherence among veterans than patients in other settings, perhaps associated with the broad access to care and follow-up services within an integrated health care system. Nonetheless, variations noted in this study highlight the need for a national effort, including within the VA, to optimize surveillance after EVAR. Future work will examine associations between different surveillance patterns and late EVAR outcomes.

## Notes

Supplement 1. **eTable 1.** Procedure codes used to identify cohort of veterans treated with EVAR **eTable 2.** Procedure codes used to identify surveillance imaging studies **eTable 3.** Method of identifying surveillance imaging studies **eTable 4.** Additional characteristics of the veteran cohort, their EVAR procedures, and their surveillance imaging **eTable 5.** Proportion of time spent in adherence by sex and age. **eTable 6.** Summary of studies evaluating imaging surveillance after EVAR **eFigure 1.** Study flowchart **eFigure 2.** First year after EVAR that veterans experience a lapse in surveillance

Supplement 2.
Data Sharing Statement
