# Supplementary material for: Imaging Surveillance Adherence After Endovascular Abdominal Aortic Aneurysm Repair at VA Hospitals
Source: JAMA Netw Open. 2025 Apr 24;8(4):e256852. doi: 10.1001/jamanetworkopen.2025.6852 (PMC12022808; doi:10.1001/jamanetworkopen.2025.6852)
Supplement: Supplement 1. — eTable 1. Procedure codes used to identify cohort of veterans treated with EVAR eTable 2. Procedure codes used to identify surveillance imaging studies eTable 3. Method of identifying surveillance imaging studies eTable 4. Additional characteristics of the veteran cohort, their EVAR procedures, and their surveillance imaging eTable 5. Proportion of time spent in adherence by sex and age. eTable 6. Summary of studies evaluating imaging surveillance after EVAR eFigure 1. Study flowchart eFigure 2. First year after EVAR that veterans experience a lapse in surveillance [file jamanetwopen-e256852-s001.pdf]

Supplemental Online Content

Newton LE, Ponukumati A, Zwain G, et al. Imaging surveillance adherence after endovascular abdominal aortic aneurysm repair at VA hospitals. *JAMA Netw Open*. 2025;8(4):e256852. doi:10.1001/jamanetworkopen.2025.6852

eTable 1. Procedure codes used to identify cohort of veterans treated with EVAR

eTable 2. Procedure codes used to identify surveillance imaging studies

eTable 3. Method of identifying surveillance imaging studies

eTable 4. Additional characteristics of the veteran cohort, their EVAR procedures, and their surveillance imaging

eTable 5. Proportion of time spent in adherence by sex and age.

eTable 6. Summary of studies evaluating imaging surveillance after EVAR

eFigure 1. Study flowchart

eFigure 2. First year after EVAR that veterans experience a lapse in surveillance

This supplemental material has been provided by the authors to give readers additional information about their work.

© 2025 Newton LE et al. *JAMA Network Open*.

eTable 1. Procedure codes used to identify cohort of veterans treated with EVAR.

Procedure code	Code type	Description
39.71	ICD09	Endovascular implantation of other graft in abdominal aorta
39.78	ICD09	Endovascular implantation of branching or fenestrated graft in aorta
04V00CZ	ICD10	Restriction of abdominal aorta with extraluminal device, open approach
04V00DJ	ICD10	Restriction of abdominal aorta with intraluminal device, temporary, open approach
04V00DZ	ICD10	Restriction of abdominal aorta with intraluminal device, open approach
04V00EZ	ICD10	Restriction of abdominal aorta with branched or fenestrated intraluminal device, one or two arteries, open approach
04V00FZ	ICD10	Restriction of abdominal aorta with branched or fenestrated intraluminal device, three or more arteries, open approach
04V03DZ	ICD10	Restriction of abdominal aorta with intraluminal device, percutaneous approach
04V03EZ	ICD10	Restriction of abdominal aorta with branched or fenestrated intraluminal device, one or two arteries, percutaneous approach
04V03FZ	ICD10	Restriction of abdominal aorta with branched or fenestrated intraluminal device, three or more arteries, percutaneous approach
04V03ZZ	ICD10	Restriction of abdominal aorta, percutaneous approach
04V04DZ	ICD10	Restriction of abdominal aorta with intraluminal device, percutaneous endoscopic approach
04V04EZ	ICD10	Restriction of abdominal aorta with branched or fenestrated intraluminal deice, one or two arteries, percutaneous endoscopic approach
04V04FZ	ICD10	Restriction of abdominal aorta with branched or fenestrated intraluminal device, three or more arteries, percutaneous endoscopic approach
04V04ZZ	ICD10	Restriction of abdominal aorta, percutaneous endoscopic approach
34701	CPT	EVAR aorto-aortic tube
34702	CPT	EVAR aorto-aortic tube, rupture
34703	CPT	Aorto-uni-iliac
34704	CPT	Aorto-uni-iliac, rupture
34705	CPT	EVAR, aortobi-iliac
34706	CPT	EVAR, aortobi-iliac, rupture
34717	CPT	Iliac branched endograft during EVAR
34841	CPT	FEVAR, aorto-aortic 1v
34842	CPT	FEVAR, aorto-aortic 2v
34843	CPT	FEVAR, aorto-aortic 3v
34844	CPT	FEVAR, aorto-iliac 4v
34845	CPT	FEVAR, aorto-iliac 1v
34846	CPT	FEVAR, aorto-iliac 2v
34847	CPT	FEVAR, aorto-iliac 3v
34848	CPT	FEVAR, aorto-iliac 4v
34802	CPT	Endo bifurcated module 2-piece
34803	CPT	Endo bifurcated module 3-piece
34805	CPT	Aorto-uni-iliac or aorto-unifemoral
34800	CPT	Endo tube
34804	CPT	Endo bifurcated unibody
34825	CPT	Endo extension, 1st vessel abdominal
34826	CPT	Endo extension, ea. add'l vessel abdominal

ICD = International Classification of Diseases; CPT = Current Procedural Terminology; EVAR = endovascular aortic repair; FEVAR = fenestrated endovascular aortic repair

© 2025 Newton LE et al. *JAMA Network Open*.

eTable 2. Procedure codes used to identify surveillance imaging studies.

Procedure code	Code type	Description	+/- Contrast
72191	CPT	CT scan	Y
72192	CPT	CT scan	N
72193	CPT	CT scan	Y
72194	CPT	CT scan	Y
72195	CPT	CT scan	N
72196	CPT	CT scan	Y
72197	CPT	CT scan	Y
72198	CPT	CT scan	Y
74150	CPT	CT scan	N
74151	CPT	CT scan	U
74152	CPT	CT scan	U
74153	CPT	CT scan	U
74154	CPT	CT scan	U
74155	CPT	CT scan	U
74156	CPT	CT scan	U
74157	CPT	CT scan	U
74158	CPT	CT scan	U
74159	CPT	CT scan	U
74160	CPT	CT scan	Y
74161	CPT	CT scan	U
74162	CPT	CT scan	U
74163	CPT	CT scan	U
74164	CPT	CT scan	U
74165	CPT	CT scan	U
74166	CPT	CT scan	U
74167	CPT	CT scan	U
74168	CPT	CT scan	U
74169	CPT	CT scan	U
74170	CPT	CT scan	Y
74171	CPT	CT scan	U
74172	CPT	CT scan	U
74173	CPT	CT scan	U
74174	CPT	CT scan	Y
74175	CPT	CT scan	Y
74176	CPT	CT scan	N
74177	CPT	CT scan	Y
74178	CPT	CT scan	Y
74179	CPT	CT scan	U
776635	CPT	CT scan	U
93975	CPT	Duplex US	N
93976	CPT	Duplex US	N
93977	CPT	Duplex US	N
93978	CPT	Duplex US	U
74181	CPT	MRI	N
74182	CPT	MRI	Y
74183	CPT	MRI	Y
74184	CPT	MRI	U
74185	CPT	MRI	Y

CPT = Current Procedural Terminology; CT = computed tomography; US = ultrasound; MRI = magnetic resonance imaging; Y = yes, N = no, U = unknown

© 2025 Newton LE et al. *JAMA Network Open*.

eTable 3. Method of identifying surveillance imaging studies, in **A.** Corporate Data Warehouse (CDW) (VA), in **B.** Centers for Medicare and Medicaid Services (CMS), and in **C.** Community Care.

A. **CDW (VA):** Surveillance imaging studies were identified in Radiology Exam, Outpatient Procedure and Inpatient Current Procedural Terminology (CPT) Procedure, and Consult domains for all VA stations.

**CDW Data Source**	**Number of unique records identified**
Outpat_VProcedure_EHR, Inpat_InpatientCPTProcedure_EHR	277,192
Rad_RadiologyExam	689,212
Outpat_VProcedure	178,769
Con_Consult	11,580

B. **CMS:** Patients identified using Veterans Health Administration (VHA) data were linked to Medicare claims data. Surveillance imaging procedures were identified using CPT codes in the carrier line and outpatient and inpatient revenue files using Healthcare Common Procedure Coding System (HCPCS) codes.

**CMS File**	**Number of unique records identified**
Carrier	23,003
Outpatient	1,724
Inpatient	<11

C. **Community Care:** The Community Care data includes Fee, Program Integrity Tool (PIT), and (Integrated veteran Care Consolidated Data Set (IVC CDS) data. Standard operating procedures from Centralized Interactive Phenomics Resource (CIPHER) were used to find CPT and ICD codes in the Community Care data sources.

**CDW Data Source**	**Number of unique records identified**
Fee	9,500
PIT	13
IVC CDS	10,032

© 2025 Newton LE et al. *JAMA Network Open*.

eTable 4. Additional characteristics of the veteran cohort, their EVAR procedures, and their surveillance imaging.

**Characteristics**	**Veterans** *n*=**27,792 (%)**
VISN (Veteran Integrated Service Network)[a]	
1: VA New England Healthcare System	1336 (4.8)
2: New York/New Jersey VA Health Care Network	1886 (6.8)
4: VA Healthcare – VISN 4	919 (3.3)
5: VA Capitol Health Care Network	918 (3.3)
6: VA Mid-Atlantic Health Care Network	954 (3.4)
7: VA Southeast Network	1892 (6.8)
8: VA Sunshine Healthcare Network	2550 (9.2)
9: VA MidSouth Healthcare Network	1124 (4.0)
10: VA Healthcare System	2688 (9.7)
12: VA Great Lakes Health Care System	1519 (5.5)
15: VA Heartland Network	1098 (4.0)
16: South Central VA Health Care Network	1994 (7.2)
17: VA Heart of Texas Health Care Network	1438 (5.2)
19: Rocky Mountain Network	1092 (3.9)
20: Northwest Network	852 (3.1)
21: Sierra Pacific Healthcare Network	1818 (6.5)
22: Desert Pacific Healthcare Network	2199 (7.9)
23: Sierra Pacific Network	1515 (5.5)
Year of EVAR[b] procedure	
2000	48 (0.1)
2001	354 (1.3)
2002	462 (1.7)
2003	477 (1.7)
2004	581 (2.1)
2005	754 (2.7)
2006	882 (3.2)
2007	1089 (3.9)
2008	1171 (4.2)
2009	1354 (4.9)
2010	1429 (5.1)
2011	1502 (5.4)
2012	1608 (5.8)
2013	1672 (6.0)
2014	1706 (6.1)
2015	1649 (5.9)
2016	1701 (6.1)
2017	1718 (6.2)
2018	1589 (5.7)
2019	1359 (4.9)
2020	1126 (4.1)
2021	1248 (4.5)
2022	1100 (4.0)
2023	1213 (4.4)
Year individual patient entered imaging cohort	
None	1055 (3.8)
2000	25 (0.1)

© 2025 Newton LE et al. *JAMA Network Open*.

2001	239 (0.9)
2002	368 (1.3)
2003	423 (1.5)
2004	516 (1.9)
2005	663 (2.4)
2006	834 (3.0)
2007	995 (3.6)
2008	1115 (4.0)
2009	1289 (4.6)
2010	1358 (4.9)
2011	1441 (5.2)
2012	1586 (5.7)
2013	1620 (5.8)
2014	1666 (6.0)
2015	1633 (5.9)
2016	1660 (6.0)
2017	1615 (5.8)
2018	1630 (5.9)
2019	1374 (4.9)
2020	1100 (4.0)
2021	1260 (4.5)
2022	1113 (4.0)
2023	1214 (4.4)

[a] Veterans Integrated Services Networks (VISNs) – Veterans Health Administration (https://www.va.gov/HEALTH/visns.asp)
[b] EVAR = endovascular abdominal aortic aneurysm repair

© 2025 Newton LE et al. *JAMA Network Open*.

eTable 5. Mean proportion of time spent in compliance, by sex and age.

Category	Proportion of time in compliance % (SD)
**Overall**, *n*=27,792	71.1% (28.6%)
**Female**, *n*=168	70.3% (31.7%)
**Male**, *n*=27,624	71.1% (28.6%)
**Age, in years**	
<65, *n*=5271	68.2% (28.3%)
65-69, *n*=7011	71.6% (27.7%)
70-74, *n*=6587	72.2% (28.5%)
75-80, *n*=4726	72.1% (29.2%)
>80, *n*=4197	71.0% (30.0%)

© 2025 Newton LE et al. *JAMA Network Open*.

eTable 6. Summary of studies evaluating imaging surveillance after EVAR.

Author	Year	Sample Size	Data Source	Date Range Studied	Design and Compliance Measure	Findings
Newton	2024	27,792	Veterans Affairs databases	2000-2023	- Proportion of time spent in surveillance compliance	- Surveillance rates overall 75%<br>- Initially high (90% at 1 year), but decrease farther from EVAR (48.9% at year 7)<br>- Factors associated with poor compliance: unmarried status, non-White race, and distance from hospital
Wanken[1]	2020	9723	Vascular Quality Initiative	2003-2015	- Kaplan-Meier survival analysis assessed freedom from surveillance failure, defined as any 15-month period in which a surveillance imaging study was not obtained	- 50% of patients experienced a surveillance failure by 4.19 years, 75% failure by 6.35 years<br>- Factors associated with surveillance failure: age >85 years, dual Medicare/Medicaid eligibility, chronic kidney disease, dementia, Northeast and Northwest regions of U.S.
Garg[2]	2015	9695	Medicare claims	2002-2005 EVAR, images through 2011	- Complete surveillance = at least 1 CT or US every 15 months<br>- Incomplete surveillance = gaps >15 months between consecutive images	- 43% of patients had complete surveillance (median follow-up 6.1 years)<br>- Factors associated with incomplete surveillance: Medicaid eligibility, low-volume hospitals, ruptured aneurysm
Schanzer[3]	2015	19,962	Medicare 20% sample	2001-2008 EVAR, images through 2010	- Lost to follow-up = no abdominal imaging study within their last 2 years of follow-up	- 50% loss to follow-up at 5 years<br>- Factors associated with loss to follow-up: age >85 years, urgent/emergent intact aneurysm or ruptured aneurysm, chronic disease, South and West regions of U.S.
Kret[4]	2013	204	Single institution	2004-2011	- Lost to follow-up = >1 year elapsed since last documented surveillance imaging<br>- Kaplan-Meier analyses to determine median follow-up	- 56% lost to follow-up (median follow-up 28 ± 10.5 months<br>- 11% with no surveillance after initial hospitalization<br>- No comorbidities or socioeconomic factors found to significantly affect follow-up
Jones[5]	2007	310	Single institution	1999-2005	- Follow-up compliance classified as either frequent (0-1 missed scheduled appointment) or incomplete (2+ missed scheduled appointments)	- Mean follow-up better in frequent group (34.7 ± 22 months) vs incomplete group (18.8 ± 18.6 months)<br>- 5-year survival, reintervention rate, and endoleak incidence similar between groups<br>- Increased major adverse events (defined as requiring urgent surgical intervention) in incomplete group
Leurs[6]	2005	4433	EUROSTAR registry	1996-2004	- Group A = attended all scheduled visits<br>- Group B = missed 1 or more visits	- 35% of patients attended all visits<br>- Factors associated with attending all visits: smoking, hyperlipidemia, unfit for open surgery or general anesthesia<br>- Complications (endoleaks, kinking, graft migration) more frequent in Group A

References for studies included in table:

© 2025 Newton LE et al. *JAMA Network Open*.

1. Wanken ZJ, Trooboff SW, Gladders B, et al. Characterization of Endovascular Abdominal Aortic Aneurysm Repair Surveillance in the Vascular Quality Initiative. *Circulation*. 2020;141(10). doi:10.1161/CIRCULATIONAHA.119.043625
2. Garg T, Baker LC, Mell MW. Adherence to postoperative surveillance guidelines after endovascular aortic aneurysm repair among Medicare beneficiaries. *J Vasc Surg*. 2015;61(1). doi:10.1016/j.jvs.2014.07.003
3. Schanzer A, Messina LM, Ghosh K, et al. Follow-up compliance after endovascular abdominal aortic aneurysm repair in Medicare beneficiaries. *J Vasc Surg*. 2015;61(1). doi:10.1016/j.jvs.2014.06.006
4. Kret MR, Azarbal AF, Mitchell EL, Liem TK, Landry GJ, Moneta GL. Compliance with long-term surveillance recommendations following endovascular aneurysm repair or type B aortic dissection. *J Vasc Surg*. 2013;58(1). doi:10.1016/j.jvs.2012.12.046
5. Jones WB, Taylor SM, Kalbaugh CA, et al. Lost to follow-up: A potential under-appreciated limitation of endovascular aneurysm repair. *J Vasc Surg*. 2007;46(3). doi:10.1016/j.jvs.2007.05.002
6. Leurs LJ, Laheij RJF, Buth J. What determines and are the consequences of surveillance intensity after endovascular abdominal aortic aneurysm repair? *Ann Vasc Surg*. 2005;19(6). doi:10.1007/s10016-005-7751-2

© 2025 Newton LE et al. *JAMA Network Open*.

eFigure 1. Flow diagram showing cohort assembly of veterans undergoing EVAR at VA hospitals from 1/1/2000-12/31/2023. EVAR = endovascular abdominal aortic aneurysm repair; OAR = open aneurysm repair.

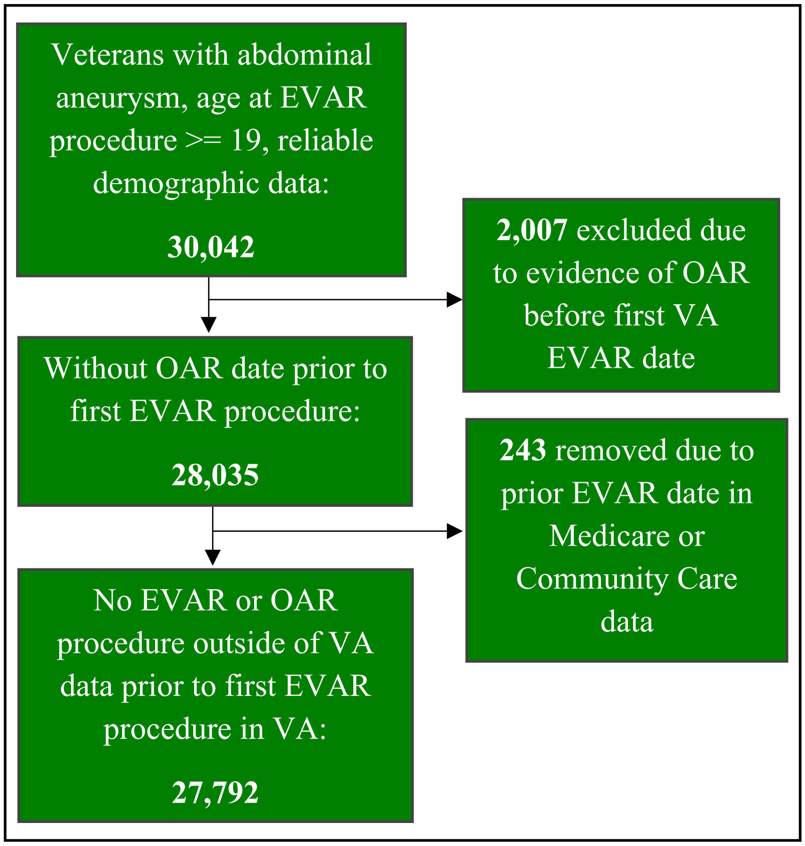

© 2025 Newton LE et al. *JAMA Network Open*.

eFigure 2. The first year after EVAR that veterans experience a lapse in surveillance.

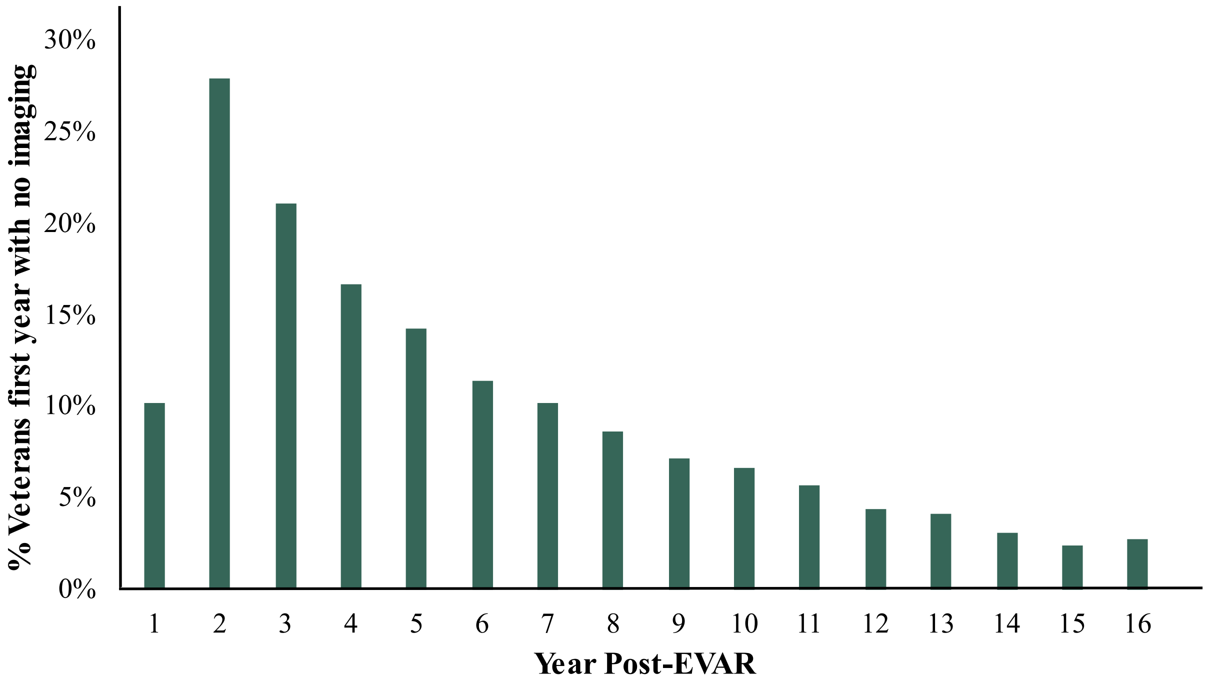

© 2025 Newton LE et al. *JAMA Network Open*.
