# Supplementary material for: Imaging Surveillance Adherence After Endovascular Abdominal Aortic Aneurysm Repair at VA Hospitals
Source: JAMA Netw Open. 2025 Apr 24;8(4):e256852. doi: 10.1001/jamanetworkopen.2025.6852 (PMC12022808; doi:10.1001/jamanetworkopen.2025.6852)
Supplement: Supplement 2. — Data Sharing Statement [file jamanetwopen-e256852-s002.pdf]

Data Sharing Statement

Newton. Imaging Surveillance Adherence After Endovascular Abdominal Aortic Aneurysm Repair at VA Hospitals. *JAMA Netw Open*. Published online April 24, 2025. doi:10.1001/jamanetworkopen.2025.6852

Data
Data available: No

Additional Information
Explanation for why data not available: The United States Department of VA regulations and ethics agreements require that the analytic data sets used for this study not leave the VA firewall without a Data Use Agreement. This limitation is consistent with other studies based on VA data. However, VA data are made freely available to researchers with an approved VA study protocol. For more information, please visit https://www.virec.research.va.gov.
